# Physician practices related to use of BMI-for-age and counseling for childhood obesity prevention: A cross-sectional study

**DOI:** 10.1186/1471-2296-12-80

**Published:** 2011-08-03

**Authors:** Holly R Wethington, Bettylou Sherry, Barbara Polhamus

**Affiliations:** 1 Division of Nutrition, Physical Activity, & Obesity, Centers for Disease Control & Prevention, 4770 Buford Hwy, NE Mail Stop K-25, Atlanta, GA 30341, USA

## Abstract

**Background:**

Screening for obesity and providing appropriate obesity-related counseling in the clinical setting are important strategies to prevent and control childhood obesity. The purpose of this study is to document pediatricians (PEDs) and general practitioners (GPs) with pediatric patients use of BMI-for-age to screen for obesity, confidence in explaining BMI, access to referral clinics, and characteristics associated with screening and counseling to children and their caregivers.

**Methods:**

The authors used 2008 DocStyles survey data to examine these practices at every well child visit for children aged two years and older. Counseling topics included: physical activity, TV viewing time, energy dense foods, fruits and vegetables, and sugar-sweetened beverages. Chi-square tests were used to examine differences in proportions and logistic regression to identify characteristics associated with screening and counseling.

**Results:**

The final analytic sample included 250 PEDs and 621 GPs. Prevalence of using BMI-for-age to screen for obesity at every well child visit was higher for PEDs than GPs (50% vs. 22%, χ2 = 67.0, p ≤ 0.01); more PEDs reported being very/somewhat confident in explaining BMI (94% vs. GPs, 87%, p < 0.01); more PEDs reported access to a pediatric obesity specialty clinic for referral (PEDs = 65% vs. GPs = 42%, χ2 = 37.5, p ≤ 0.0001).

In general, PEDs reported higher counseling prevalence than GPs. There were significant differences in the following topics: TV viewing (PEDs, 79% vs. GPs, 61%, χ2 = 19.1, p ≤ 0.0001); fruit and vegetable consumption (PEDs, 87% vs. GPs, 78%, χ2 = 6.4, p ≤ 0.01). The only characteristics associated with use of BMI for GPs were being female (OR = 2.3, 95% CI = 1.5-3.5) and serving mostly non-white patients (OR = 1.8, 95% CI = 1.1-2.9); there were no significant associations for PEDs.

**Conclusions:**

The findings for use of BMI-for-age, counseling habits, and access to a pediatric obesity specialty clinic leave room for improvement. More research is needed to better understand why BMI-for-age is not being used to screen at every well child visit, which may increase the likelihood overweight and obese patients receive counseling and referrals for additional services. The authors also suggest more communication between PEDs and GPs through professional organizations to increase awareness of existing resources, and to enhance access and referral to pediatric obesity specialty clinics.

## Background

Obesity in youth is a significant public health problem; almost 17% of children and adolescents 2-19 years are obese based on 2007-08 estimates [1]. To address overweight and obesity, the American Academy of Pediatrics (AAP), the US Preventive Services Task Force, and the Expert Committee on Childhood Obesity, comprised of representatives from professional organizations, experienced scientists and clinicians, recommend using BMI-for-age to screen for overweight and obesity in children ages two years and over, and also counseling on behaviors such as nutrition and physical activity [2-5]. Primary care providers with pediatric patients are well suited to screen for overweight and obesity and counsel on health-related behaviors; however, previous research has shown primary care providers are not routinely screening for overweight and obesity using BMI-for-age [6,7].

Using BMI-for-age increases the likelihood that pediatricians will identify overweight or obesity status in youth [6,8]. Furthermore, of all overweight or obese youth, those identified as overweight or obese were more likely to receive nutrition and physical activity counseling [6,9,10] and appropriate treatment [11,12]. Identifying overweight or obese children and adolescents is important because they are at an increased risk for several cardiovascular disease risk factors and type II diabetes compared to normal weight children and adolescents [13,14]. Furthermore, childhood obesity can increase the likelihood of adult obesity [15,16].

The purpose of the study was to determine the proportion of pediatricians (PEDs) and general practitioners (GPs) with pediatric patients who 1) screen for obesity using BMI-for-age at every well child visit; 2) are confident in explaining BMI-for-age results to children and their parents, 3) have access to a pediatric obesity specialty clinic; and 4) counsel on physical activity, TV viewing time, intake of energy dense foods (i.e., the amount of energy (kilocalories or kcal) in a gram (g) of food [17]; foods with lower energy density such as raw carrots have fewer kilocalories per gram than those with higher energy density, such as French fries), fruits and vegetables, and sugar-sweetened beverages. The investigators tested for differences in use of BMI-for-age and counseling habits between PEDs and GPs. It was hypothesized that PEDs would screen and counsel more than GPs, thus differences may indicate a need for general or family practice residency programs or continuing medical education to address this issue. The investigators also examined whether there were differences in the use of BMI-for-age as a screening tool and counseling habits by child's weight status because physicians may be more inclined to counsel children who appear to be overweight or obese. In addition, an exploratory analysis was conducted to examine predictors potentially associated with physicians' likelihood of screening for obesity using BMI-for-age and providing counseling in order to identify those who are following recommended protocol.

## Methods

This study is based on data from the DocStyles 2008 web-based survey. DocStyles is a web-based panel survey developed by Porter Novelli, with input from federal public agencies as well as other profit and non-profit organizations. The survey instrument was designed to provide insights into physicians' attitudes and counseling behaviors regarding a variety of health issues relevant to adult or pediatric patients that included but were not limited to pregnancy health, cancer screenings, nutrition, physical activity, and weight status. The CDC Human Subjects Review determined these analyses were exempt from Human Subjects Review because this is a secondary data analysis using data without identifiers.

### Participants

This study is based on PEDs and GPs, who comprise part of the DocStyles 2008 data. The sample originated from the Epocrates Honor Panel (http://www.epocrates.com), which consists of 135,000 verified physicians from multiple specialties invited to participate in surveys [18]. The goal for DocStyles was 250 PEDs and 1,000 GPs respondents. In order to reach this goal, Porter Novelli employed a probability sampling method to randomly select a sample of 14,346 physicians from the Epocrates Honors Panel database to match the American Medical Association (AMA) files in terms of name, age, sex, and region [19]. Of these 14,346, 2,207 are PEDs and 7,205 are GPs. A total of 1,785 PEDs and 5,671 GPs did not respond to the invitation or tried to respond after the survey closed; 146 PEDs and 457 GPs logged in to take the survey but were eliminated due to filled quotas for their specialty; one PED and 11 GPs did not complete the entire survey; and 22 PEDs and 72 GPs were eliminated based on the screener questions. Potential participants were screened at the beginning of the survey to assure they met the following criteria: 1) currently practicing in the US; 2) working in an individual, group, or hospital practice; and 3) have been practicing medicine for at least three years. Response rates, 19% for PEDs and 21% for GPs, were calculated by weighting respondents who were terminated due to filled quotas as a factor of the overall sample pool as opposed to classifying them as standard incompletes [19]. The different physician specialties were included because they were of particular interest to the data collectors and the total sample by itself is not intended to be representative of a national population of physicians or physician specialties. There are no weights in the sample, rendering it impossible to control for type of physician. Participating physicians received an honorarium of $50-$75 for their time and were not required to participate in the survey and could opt out at anytime. The study was limited to include all PEDs (n = 250) and GPs with pediatric patients (n = 621).

Porter Novelli compared the overall DocStyles sample, by physician specialty, to the AMA master file for gender, age, and region of the country. For GPs, a slightly higher percentage of males were included in the overall DocStyles sample compared to the AMA master file. For PEDs, 61% of DocStyles respondents were male while the AMA master file shows 44% of PEDs are male. The authors were unable to assess the comparability of this analytic sample of GPs to the AMA master file because sample was restricted to only those who see pediatric patients. The sample protocol for DocStyles is complex and although there are substantial efforts to assure representativeness it is possible that volunteer selection bias is present.

### Analysis I. Prevalence of Screening, Confidence, Referral, and Counseling

The first analysis examined these on the basis of the following questions:

1) "How often do you use BMI-for-age to screen for obesity for children 2 years of age or older? (*Never, Rarely, At some visits, At most visits, or At every well child visit*)."

Because it is recommended physicians use BMI-for-age to screen for obesity at every well child visit, the responses were dichotomized to reflect physicians who do and do not use BMI-for-age to screen for obesity at every well child visit.

2) "How confident are you in explaining BMI-for-age results to children and their parents? (*Not at all confident, Slightly confident, Somewhat confident, very confident*)."

Responses were collapsed into two categories: *Somewhat *or *Very confident *and *Not at all *or *Slightly confident*.

3) "For your obese patients with complications or co-morbidities, do you have access to a pediatric obesity specialty clinic (typically a tertiary care center) for referral? (*Yes *or *No*)."

4) for each of the following topics: being physically active, amount of TV time, consumption of energy dense foods, eating fruits and vegetables daily, and consumption of sugar-sweetened beverages respondents needed to indicate whether they or a staff member discussed the topic with all patients, with both those overweight (BMI 85^th ^-94^th ^percentile) and those obese (BMI > 95^th ^percentile), only with those overweight (BMI 85^th^-94^th ^percentile), only those obese (BMI > 95^th ^percentile), or they generally did not discuss it. The question was presented in tabular form. The responses were collapsed into the following categories: *All patients and Only with overweight or obese.*

### Analysis 2: Predictors of Screening and Counseling

To predict use of BMI-for-age to screen for obesity and physicians' counseling habits on prevention the authors used the same predictor variables for both types of physicians: physicians' gender; physicians' race/ethnicity (non-Hispanic white or Other); number of years practiced (< 10 years or ≥ 10 years); physicians' type of practice (individual or group/hospital/clinic); patients' race/ethnicity (mostly white or mostly minority); and patients' income category (low or middle/upper).

### Statistical Analysis

Analyses were conducted with SAS version 9.2 [20]. For Analysis 1, the authors used chi-square tests to determine differences in screening, confidence, and referral between the two physician groups. Next, the authors determined the prevalence among physicians who responded that they had "generally discussed" that topic with all patients, "only discuss that topic with overweight or obese patients", or they "generally do not discuss that topic with any patient" overall and for each topic area individually; prevalences are reported for each physician type and the corresponding differences in proportions based on chi-square test using a 0.05 level of significance. For Analysis 2, adjusted logistic regression to determine predictors of using BMI-for-age at every well child visit versus not using BMI-for-age at every well child visit was conducted. Adjusted logistic regression was conducted to determine predictors of counseling all patients versus only overweight/obese patients for each counseling topic to investigate if physicians were more likely to report increased counseling for overweight/obese patients. Analyses were run separately for each physician type.

## Results

Demographic characteristics of the study participants are presented in Table 1. For PEDs and GPs, more respondents were male (63% and 76%, respectively). The majority of respondents were non-Hispanic white (66% PEDs, 75% GPs) and had been practicing for 10 years or more (60% PEDs, 61% GPs). Further, the majority of PEDs (60%) and GPs (66%) were in a group practice compared to an individual practice or hospital/clinic-based practice.

**Table 1:** Demographics of Pediatricians and General Practitioners, DocStyles, 2008

	PEDs* (n = 250) n (%)	GPs† (n = 621) n (%)	*χ2*	*p *value
Gender			13.8	≤ 0.001

Male	158 (63)	470 (76)		

Female	92 (37)	151 (24)		

Race/Ethnicity			7.0	≤ 0.01

Non-Hispanic white	166 (66)	466 (75)		

Other	84 (34)	155 (25)		

Years of Practice			0.08	0.8

< 10 Years	100 (40)	242 (39)		

≥ 10 Years	150 (60)	379 (61)		

Type of Practice			12.8	≤ 0.001

Individual	25 (10)	125 (20)		

Group/Hospital/Clinic	225 (90)	496 (80)		

* PEDs = Pediatricians

†GPs = General Practitioners with pediatric patients.

### Analysis I. Prevalence of Screening, Confidence, Referral, and Counseling

Overall, 30% of physicians reported screening for obesity using BMI-for-age at every well child visit (data not shown). Rates were higher for PEDs than GPs (50% vs. 22%, p ≤ 0.01) (Table 2). Similarly, PEDs were more likely to respond that they were somewhat or very confident in explaining BMI-for-age results compared to GPs (PEDs, 94% vs. GPs, 87%, p ≤ 0.01). Although there were differences, 87% or more reported having "confidence explaining BMI-for-age to patients and their parents". Access to a pediatric obesity specialty clinic for referral was reported by 65% of PEDs, compared to 42% of GPs (p ≤ 0.0001).

**Table 2:** Obesity Screening, Confidence, and Referral to Pediatric Obesity Specialty Clinic by Physician Type, DocStyles, 2008

	PEDs* n = 250 n (%)	GPs† n = 621 n (%)	*χ2*	*p *value
**Use BMI-for-age for obesity screening**			67.0	p ≤ 0.0001

At every well child visit	126 (50)	138 (22)		

Not at every visit	124 (50)	483 (78)		

**Confident explaining BMI results**			9.7	p ≤ 0.01

Somewhat or very confident	235 (94)	538 (87)		

Slightly or not at all confident	15 (6)	83 (13)		

**For obese patients with complications or co-morbidities, do you have access to a pediatric obesity specialty clinic?**			37.5	p ≤ 0.0001

Yes	162 (65)	260 (42)		

No	88 (35)	361 (58)		

*PEDs = Pediatricians

†GPs = General Practitioners with pediatric patients.

Overall, it was found that 52% of PEDs and 45% of GPs reported counseling all patients on all topic areas and 6% of PEDs and 11% of GPs reported counseling only overweight or obese patients on all topic areas (Table 3). In general, for each topic area, most PEDs and GPs reported counseling all patients, while a smaller proportion of PEDs and GPs reported counseling only overweight or obese patients. Only a few physicians reported that they generally do not discuss one or more counseling issues Counseling all patients was highest for fruit and vegetable consumption and physical activity and lowest for consumption of energy dense foods. Excluding those who did not discuss, PEDs had a significantly higher prevalence of counseling all patients than GPs for TV viewing time (PEDs, 79% vs. GPs, 61%, χ2 = 19.1, p ≤ 0.0001) and for consumption of fruits and vegetables PEDs, 87% vs. GPs, 78%, χ2 = 6.4, p ≤ 0.01) (data not shown).

**Table 3:** Physician Self-reported Counseling Practices by Physician Type, DocStyles, 2008

	PEDs* n = 250 n (%)	GPs† n = 621 n (%)
**Overall Counseling Practices**‡		

All patients counseled on all topic areas	130 (52)	282 (45)

Only overweight or obese patients counseled on all topic areas	14 (6)	67 (11)

Patients not counseled on any topic area	2 (1)	6 (1)

**Physical activity**		

All patients	208 (83)	487 (78)

Only with overweight or obese	40 (16)	126 (20)

Generally do not discuss	2 (< 1)	8 (1)

**TV viewing time**		

All patients	197 (79)	377 (61)

Only with overweight or obese	46 (18)	196 (32)

Generally do not discuss	7 (3)	48 (8)

**Consumption of energy dense foods**		

All patients	147 (59)	344 (55)

Only with overweight or obese	100 (40)	263 (42)

Generally do not discuss	3 (1)	14 (2)

**Consumption of fruits and vegetables**		

All patients	217 (87)	487 (78)

Only with overweight or obese	30 (12)	117 (19)

Generally do not discuss	3 (1)	17 (3)

**Consumption of sugar-sweetened beverages**		

All patients	178 (71)	409 (66)

Only with overweight or obese	69 (28)	206 (33)

Generally do not discuss	3 (1)	6 (1)

* PEDs = Pediatricians

†GPs = General Practitioners with pediatric patients

‡ Percentages do not add to 100% because not all respondents reflected in results.

### Analysis 2: Predictors of Screening and Counseling

Table 4 reports predictors for using BMI-for-age to screen for obesity. Only two predictors were found among GPs: female GPs were more likely to report screening children at every well child visit compared to male GPs (OR = 2.3, 95% CI = 1.5, 3.5) and GPs with a patient population that is mostly non-white were more likely to report screening children at every well child visit compared to GPs with a mostly white patient population (OR = 1.8, 95% CI = 1.1, 2.9). No predictors were significantly associated with BMI screening among PEDs.

**Table 4:** Adjusted Odds Ratios for BMI-for-Age at Every Well-Child Visit by Physician Type, DocStyles, 2008*

	PEDs † n = 250				GPs ‡ n = 621			
	**At every visit n (%)**	**Not at every visit n (%)**	**OR**	**95% CI**	**At every visit n (%)**	**Not at every visit n (%)**	**OR**	**95% CI**

**Physician Characteristics**								

**Gender of Physician**								

Male	75 (48)	83 (53)	1.0		85 (18)	385 (82)	1.0	

Female	51 (55)	41 (45)	1.4	0.8, 2.3	53 (35)	98 (65)	2.3	1.5, 3.5§

**Race of Physician**								

Non-Hispanic White	86 (52)	80 (48)	1.0		101 (22)	365 (78)	1.0	

Other	40 (48)	44 (52)	0.8	0.5, 1.4	37 (24)	118 (76)	0.9	0.6, 1.5

**Years of Practice**								

< 10 Years	51 (51)	49 (49)	1.0		64 (27)	178 (74)	1.0	

≥ 10 Years	75 (50)	75 (50)	1.0	0.6, 1.6	74 (20)	305 (81)	0.7	0.5, 1.1

**Type of Practice**								

Individual	12 (48)	13 (52)	1.0		27 (22)	98 (78)	1.0	

Group/Hospital/Clinic	114 (51)	111 (49)	1.1	0.5, 2.5	111 (22)	385 (78)	0.9	0.6, 1.5

**Patient Characteristics**								

**SES of Patients**								

Lower SES	81 (50)	82 (50)	1.0		84 (24)	266 (76)	1.0	

Middle-Upper SES	45 (52)	42 (48)	1.1	0.6, 2.0	54 (20)	217 (80)	1.0	0.6, 1.5

**Race/Ethnicity of Patient Population**								

Mostly white	76 (50)	76 (50)	1.0		97 (20)	393 (80)	1.0	

Mostly non-white	50 (51)	48 (49)	1.1	0.6, 2.0	41 (31)	90 (69)	1.8	1.1, 2.9§

* Both models adjusted for gender of physician, years of practice, race of physician, type of practice, SES of patients, and race of patient population

† PEDs = Pediatricians

‡ GPs = General Practitioners with pediatric patients

§ Significant because confidence interval does not include 1.0.

Predictors varied by topic and generally were not significantly associated with counseling with three exceptions (data not shown). PEDs with a race/ethnicity of "Other" were more likely to counsel all patients on energy dense foods compared to non-Hispanic white PEDs (OR = 1.9, 95% CI = 1.1, 3.5). Female GPs were more likely to counsel all patients on TV viewing time (OR = 1.9, 95% CI = 1.2, 2.9) and fruit and vegetable consumption (OR = 2.0, 95% CI = 1.2, 3.4) compared to male GPs.

## Discussion

This study documented that only 50% of PEDs and 22% of GPs who treated pediatric patients reported routinely using BMI-for-age to screen for weight status in all patients at each well child visit as recommended by the AAP [3]. While the authors' hypothesis that PEDs would use BMI-for-age more than GPs was supported, these findings suggest that increased efforts are needed to attain the AAP goal among both specialties.

The literature documents fairly similar levels of use of BMI by PEDs compared to GPs. This study's finding that 50% PEDs reported using BMI-for-age at every well child visit is slightly higher than previous findings, which ranged from 11% to 35% for reporting always or generally using BMI [6,21-24]. However, these results are very similar to a 2010 AAP study that found 52% of PEDs compute or plot BMI at most or every well child visit [25]. This study's finding of 22% of GPs reporting use of BMI-for-age to screen for obesity at every well child visit, although disconcerting, is consistent with previous studies. Woolford and colleagues (2008) reported 17% of family physicians' used BMI charts [21] and Kolagotla and Adams' (2004) found that 22% of family physicians routinely used BMI on pre-adolescents [22]. Interestingly, Kolagotla and Adams, who reported results by patient age, found that 5% of family practitioners routinely used BMI for children ages 3-7 years, and 49% routinely used BMI for adolescents. The authors were unable to examine whether GPs had different levels of BMI-for-age usage for different age groups. This is an area in need of further investigation.

Both PEDs and GPs reported high levels of confidence in explaining BMI-for-age results, although a significantly higher proportion of PEDs reported a high level of confidence. For both specialties, these findings suggest that factors other than lack of confidence may be responsible for the low levels of using BMI-for-age, such as time [26,27].

A significantly lower proportion of GPs with pediatric patients reported access to a pediatric obesity specialty clinic than PEDs. This highlights a potential disparity for GPs with pediatric patients and could be one explanation as to why a smaller proportion of GPs screen with BMI-for-age at every visit compared to PEDs: GPs do not have a sufficient protocol for their obese pediatric patients. GPs should be encouraged to access AAP resources in their states and communities to help them find referral clinics for their obese patients. Further, organizations such as AAP could include outreach efforts to GPs with pediatric patients.

The five counseling topics were examined separately because they are five of the six priority target behaviors to prevent and control obesity for the Division of Nutrition, Physical Activity, and Obesity at the Centers for Disease Control and Prevention (http://www.cdc.gov/nccdphp/DNPAO/aboutus/index.html). The counseling topics were also included in the recommendations for healthcare providers' counseling for pediatric patients and their families by the Expert Committee on Childhood Obesity [4].

This study assessed if PEDs and GPs were counseling on a topic area in general, not if PEDs' and GPs' were educating patients and their parents on specific recommendations. Recommendations exist for children on three of the five counseling topics: physical activity, TV viewing time, and fruit and vegetable consumption. Regarding physical activity, the 2008 Physical Activity Guidelines for Americans recommend children and adolescents (ages 6-17 years) engage in 60 minutes or more of physical activity daily, where most of the 60 minutes or more per day be either moderate- or vigorous-intensity and include vigorous-intensity physical activity at least three days per week [28]. Furthermore, children and adolescents should engage in muscle-strengthening and bone-strengthening exercises as part of daily physical activity, or at least three days of the week [28]. Regarding TV viewing, the AAP currently recommends youth ages two years and over engage in no more than two hours of television viewing, or screen time (television plus other forms of media for entertainment purposes) per day [29]. Lastly, recommendations for fruit and vegetable exist, yet recommended consumption amounts vary depending on a child's age, sex, and activity level, where for example children aged 2 years require daily about 1 cup each of vegetables and fruit and 18 year olds require daily about 3 cups of vegetables and 2 cups of fruit [30,31].

Interestingly, this study found among PEDs that the three topic areas with recommendations have the highest prevalence of counseling: physical activity (83%), TV time (79%), and fruit and vegetable consumption (87%) compared to energy dense foods (59%) and sugar-sweetened beverages (65%). These findings are very similar to an AAP study that found 86% of PEDs reported counseling all patients on physical activity, 76% counseled on TV viewing time, 89% on fruits and vegetables, 44% on energy dense foods, and 65% on sugar-sweetened beverages [25]. The lower counseling prevalence of energy density and sugar-sweetened beverages suggests that if there were recommendations for these topic areas, physicians might counsel their patients in these areas more frequently. Consumption of energy dense foods was the least counseled topic by both PEDs and GPs. This is an important issue because of the frequent consumption of high energy dense foods, such as fast food [32,33]. Somewhat similar to PEDs, GPs with pediatric patients reported a higher prevalence of counseling all patients on physical activity and fruit and vegetable consumption compared to the other three topics examined.

It is unknown why PEDs and GPs with pediatric patients do not report higher rates of using BMI-for-age and counseling, and why a discrepancy exists between the two specialties. One barrier may be the lack of time because evidence shows that the time needed for recommended screening and counseling exceeds the available time for primary care visits [26,27]. Future research could discover other barriers that PEDs and GPs with pediatric patients confront and determine if different steps are necessary to overcome such barriers for the two different specialties.

The analyses to identify predictors associated with use of BMI-for-age and counseling habits documented that race/ethnicity of PEDs and gender of physician among GPs as significant predictors. Interestingly, "Other" PEDs were more likely to counsel all patients on energy dense foods compared to non-Hispanic white PEDs. GPs with a patient population that is mostly non-white were also more likely to use BMI-for-age. To the best of the authors' knowledge, these findings have not been previously reported. Additionally, among GPs, females were more likely to use BMI-for-age, counsel all patients on TV viewing time, and counsel all patients on fruit and vegetable consumption compared to male GPs. This is consistent with previous research showing female physicians were more likely to offer preventive services and counseling compared to male physicians [22,34,35]. These findings need to be further explored so that education and training can be targeted to those most in need of changing their screening and counseling practices.

There were two strengths to this study. First is the attempt to match the convenience sample of physicians included in the Epocrates Honors Panel to the AMA master file for age, gender, and region, for each specialty area to make the findings more generalizable. A second strength is the inquiry about a quality of care issue, the use of BMI-for-age to screen for childhood obesity, given this is the AAP recommended method for screening. Previous research has shown that substantial proportions of PEDs and family practitioners reported not using the recommended BMI-for-age to screen for obesity, but they relied on height and weight growth charts, visual assessment, evaluating trends overtime, or only calculating BMI if concerned [22,27]. With increased attention on obesity, it is important to demonstrate whether screening practices, based on the recommended tool are improving.

This study has limitations. First, there may be sampling bias. While attempts were made to match the sample to the AMA master file for age, gender, and region, there were differences in the sample for gender compared to the AMA master file. This sample included a higher percentage of male physician respondents for both PEDs and GPs compared to the AMA master file. Additionally, the sample may not be representative of all PEDs and GPs because of potential for volunteer bias due to quota sampling and the original database being an opt-in database. Generalizing results to all PEDs and GPs is not possible because of the low response rates for PEDs and GPs. A second limitation is a possible reporting bias from physicians' self-reported use of BMI-for-age to screen for obesity resulting in an overestimated BMI-for-age use. A third limitation is that the authors were not able to assess methods other than BMI-for-age for obesity screening. It is possible practitioners in this sample are using other methods to assess weight status although not the recommended protocol. Using methods other than BMI-for-age has different implications than not screening at all. For example, obese children who are not screened at all may be less likely to receive appropriate referral compared to obese children who receive appropriate referral after being diagnosed using a different method. Unfortunately, the data did not allow for more exploration for use of other methods. A fourth limitation is the authors did not specify which type of specialty clinic when asking about referral to a pediatric obesity specialty clinic. Physician respondents may have interpreted this question differently (e.g., bariatric surgery clinic, endocrinologist, lipidologist). However, those who responded affirmatively have a system in place to refer obese patients regardless of clinic type. A fifth limitation, the responses to the question about counseling activities could have been biased or incomplete because the physician respondent might not know whether or not his/her staff is counseling on overweight prevention topics that were listed in the question. Finally, the number of calculations necessary to examine counseling habits by six physician characteristics (i.e., gender of physician, years practiced, race of physician, type of practice, SES of patients, and race of patient population), five counseling topic (physical activity, TV viewing, energy dense food consumption, fruit and vegetable consumption, and sugar-sweetened beverage consumption), and two physician types (PEDs and GPs) resulted in 60 odds ratios (6*5*2). With a significance level of 0.05, this increased the possibility of a type I error.

### Recommendations

These findings suggest a great need for some important next steps to increase adherence to the AAP and Institute of Medicine (IOM) obesity screening recommendations [2,3,36], counseling recommendations on nutrition and age-appropriate physical activity, as well as establish a system for referral to a pediatric obesity specialty clinic. Strategies for improving screening include changes in the protocol for staff to screen for obesity [35] and having a nurse or assistant calculate BMI for the physician have been identified as facilitators to use of BMI [27]. Another strategy is the use of electronic medical record systems that automatically calculate BMI-for-age once height and weight data are entered [37]. This would allow the graphs to be readily available to the physician to review and draw attention to necessary counseling. Another strategy is continuing medical education (CME) for PEDs and GPs with pediatric patients which may increase self-efficacy and knowledge of obesity screening and counseling, which may in turn increase the level of confidence when discussing the topic with parents [25]. There are existing resources to increase physician screening and counseling expertise. The Maine Youth Overweight Collaborative (MYOC) and the Washington state model provide examples for improving physicians' ability to screen for obesity and offer appropriate management of obesity [38,39]. The MYOC intervention provides tools for clinical decision support and counseling and self-management support for families and patients, [38] while the Washington state model integrates obesity prevention and management into the clinic setting that emphasizes small, consistent behavior changes, and self-regulation of eating/feeding practices with children, teenagers, and families; building local community partnerships; and encouraging broader advocacy and policy change [39].

In addition, as of January 2010, the American Board of Pediatrics requires ongoing practice improvements for maintenance of certification [40]. These ongoing practice improvements ensure that board certified PEDs understand the importance of BMI-for-age to identify obesity and provide obese patients with appropriate medical assessment. In the future, the 2009 Healthcare Effectiveness Data and Information Set (HEDIS) measure, "Weight Assessment and Counseling for Nutrition and Physical Activity for Children/Adolescents," will provide useful information on whether or not healthcare providers are screening for obesity with BMI and offering appropriate counseling for children and adolescents and their parents [41].

## Conclusions

Only 50% of PEDs and 22% of GPs with pediatric patients reported using BMI-for-age, about half of PEDs and GPs reported counseling all patients on the five weight-related topics included in this survey, and only 65% of PEDs and 42% of GPs reported access to a pediatric obesity specialty clinic. More research is needed to better understand why BMI-for-age is not being used to screen at every well child visit and how frequently other screening tools are being used. This is important because previous research has shown that plotting BMI leads to greater recognition of a weight problem [23], and a higher likelihood of receiving counseling [6,9,10] and treatment [12,42]. While the findings that physicians reported counseling all patients and not just those who were overweight or obese presented here are reassuring, the overall counseling prevalence is somewhat low. Further, it remains crucial to identify overweight or obese status and ensure these patients receive appropriate counseling and physicians make referrals for additional services. The authors also suggest more communication between PEDs and GPs through professional organizations to increase awareness of existing resources, and to enhance access and referral to pediatric obesity specialty clinics. Physician practices have an important role in overcoming the epidemic of pediatric obesity.

## Competing interests

The authors declare that they have no competing interests.

## Authors' contributions

HRW participated in the design of the study, conducted data analysis and interpretation of data, and drafted the manuscript. BS participated in the design of the study, acquisition of data, interpretation of data, and revising the manuscript critically for important intellectual content. BP participated in the acquisition of data, interpretation of data, and revising the manuscript critically for important intellectual content. All authors read and approved the final manuscript.

## Pre-publication history

The pre-publication history for this paper can be accessed here:

http://www.biomedcentral.com/1471-2296/12/80/prepub
